# An Ishmaelite

**Published:** 1888-05-12

**Authors:** Leslie Keith

**Affiliations:** Author of "The Chilcotes," "Uncle Bob's Niece," "East and West," etc.


*(To be continued.)*

### AN ISHMAELITE.

By Leslie Keith,

Author of "The Chilcotes," "Uncle Bob's Niece," "East and West," etc.

### Chapter VI.—After Five Years.

On a certain day of a certain year Arthur Eastgate went up the steps of a house in Pont Street, and rang the bell. The neighbouring houses were mostly shuttered and abandoned to caretakers, and the whole region had that air of desolation Belgravia wears at the close of the season.

A man servant came tardily to the door at his second summons.

"Has Mrs. Bellynge returned to town?" Arthur asked.

"Yes, sir; she came to town last night."

"I will see her," said Arthur; "you need not announce me."

When he had opened the drawing-room door, a lady who was once Helen Eastgate, but these three years past had been Helen Bellynge, started up out of an easy chair.

"Oh, Arthur, is it you?" she cried, running forward to meet him, and holding up her face for his kiss. "I was afraid it was a visitor!"

"Well, so it is."

"Oh, but a company visitor; you don't mind our brown holland condition, do you? Those chair covers always remind me of our morning pinafores long ago. I think I feel nurse's fingers now, as she tied us up in them. Do you remember what bony knuckles she had?"

"I remember," said Arthur, looking absently about him. "And what are your plans, Nellie?"

"We are going to Scotland in a week, when Richard's business is completed. I dare say I shall be able to endure the brown holland for that space of time. Richard is more fortunate, he can dine at his club."

"For a week? Then meantime you are free?"

"Free to go and dine somewhere with you? Oh, yes, my dear boy; free and delighted, so long as the somewhere isn't Portland Place."

"I wasn't thinking of dinner," said Arthur, gravely; "I was thinking of a journey that we might take together. Do you remember what time of year it is, Nellie? Do you remember what happened five years ago?"

She shrank a little at his words, and a sudden hot blush flamed out on her pale cheeks. She was still the same pretty, timid Nellie as of old, with the frightened, wistful, dark eyes. Arthur noted those signal flags of distress, and talked on to give her courage time to reassert itself.

"You and I, his sister and brother, ought to be there to welcome him back. Think what that coming back will mean to him." He paused abruptly. "It's no good; we can't think of it, or put ourselves in his place, and we've no business to guess at his feelings; it's our part we've got to think of, and if you were there to take him by the hand—you know what I mean, Nellie, you who are a mother now with a boy of your own.

"I daren't, Arthur, I daren't," she cried, and then she hid her burning face. "Richard would never forgive me. He—he wouldn't understand, and I couldn't explain—"

"Do you mean that you never talked to Bellynge—that you never told him of Fred —"

Her head drooped lower. She lifted it a moment to look at him imploringly.

"Don't despise me, Arthur," she said. "I never told him about—about those hopes we once had before—Fred went away. What would have been the use? It was all over and done with. If I had waited—if I had been free—it would still have been no use. You said yourself we could never marry —"

"Perhaps not," said Arthur with a short, impatient sigh. Then he said more gently, "I didn't blame you for marrying, Nellie." He was very tender and gentle towards Nellie—poor, weak, foolish Nellie, but he did not expect her to be a heroine. He had that protecting, chivalrous feeling for women that the best men have, even in these days, but he had never reproached her for twining herself clingingly round the first strong support that was offered her. There might be a noble woman strong and brave enough to take the burden of Fred's disgrace upon her shoulders and help him to live it down, but little Nellie was not of this stuff. Yet he felt it was a grave mistake to have set out on her wedded life with a secret unshared by her husband.

"I was so miserable after auntie died," Nellie went on in faltering excuse. "Oh, Arthur, you can't think what it was—auntie gone and you too, and Fred where I could never see him or hear of him; and nobody but uncle, who used to frighten me with his gloomy ways. I suppose my love was not worth much," she said with sad humility, "since it could not bear the strain of that terrible loneliness—and when Richard came and seemed to care for me —"

"Poor Nellie," said Arthur, gently. "It was a good thing, too, that he came and carried you off, only you might have talked to him about Fred."

Nellie looked at him with a kind of wonder in her great dark eyes. Though she was so shy and timid, she had seen something of the world since she married, and she had learned instinctively to set her actions and opinions by its standards. "If you have a poor relation, or if you have the greater misfortune to have a relative who has stained and disgraced the family name and the family honour, it is your duty to cut him," says the world. "Other people will remember him very likely, with that keen memory other people have for our blemishes, but they will respect you the more for your silence.

Are they not silent too over those little family matters that would have such a very awkward look if they were bruited about; and is there not in every home in the kingdom a skeleton buried away from public view?" Fred was the skeleton in this Belgravian mansion, possibly, though, his presence had not made itself felt there adversely as yet. Nellie was too humble and gentle to be moulded into a cynic, but she was also too timid to combat society's verdict. She thought of Fred every day; perhaps she prayed for him when she knelt by her child's cradle; but she never spoke of him.

Richard Bellynge knew the story, of course. The scandal had been too widespread for him to miss it, and he was quite of the world's mind in holding it best to give it decent interment as speedily as possible. He held himself, indeed, to have done rather an honourable and fine thing in marrying a girl with such a blot on her family history, and he would not for a moment have tolerated any revival of the story in his own household. He was not a bad man, as men go. He was only a man of the world, who took his maxims from that teacher, and did not relish a convict for a cousin. If he could have heard Arthur's cool proposal made in his own drawing-room, very likely he would have shown that young fellow to the door, and requested him never more to darken it. Suppose that the society journals were to get hold of the suggestion, and to set it forth thus for the information of their readers:—

"We understand that the young and charming Mrs. Richard Bellynge has accompanied her brother, Mr. Arthur Eastgate, to Dartmoor, to receive and welcome her cousin, Mr. Frederic Eastgate, who is about to be set at liberty after suffering five years of penal servitude for forgery."

"Well, then, suppose it," Arthur would have cried out. "What, then? Will it kill her or you to let the world know that there is one good woman in it who is not ashamed to forgive as she hopes to be forgiven?"

Arthur's views were no doubt very visionary and impracticable, and quite unworkable; and his ideas of a man's duty towards his fellow-man as archaic as the philosophy of Marcus Aurelius, whose wisdom nobody dreams of applauding nowadays—as old and as little practised, perhaps, as the teachings of a diviner moralist than the Roman Emperor. In the years that had changed Nellie into a wife and a mother, he, too, had seen something of life; but he had never wavered from that purpose formed in his boyhood, and sealed by his aunt's dying bed, to devote himself and all he had to his cousin.

He did not press Nellie any more, and when she looked up and said with pained self-reproach: "I wish I could go," he returned cheerfully:

"Never mind, Nell, I'm going; and I dare say he'll like that best at first. By-and-by, when he has got time to grow used to things a little, you may meet."

Nellie looked very downcast. She knew that her husband would never allow that meeting if he could prevent it; but she did not wish to damp Arthur's outset with her own fears.

"You must let me see my godson," he said, looking at his watch; "it's almost time I was getting home. I set off in two days, and I don't know when I may see the youngster again."

"We left baby in the country; it was better for him. We shall pick him up on our way north. I think it is the loss of my little playmate that makes the holland pinafores seem so oppressive." She looked round on the muffled furniture with a rather forlorn smile. "But you will come to us when you return, Arthur? We shall be here till the end of next week."

"I don't know when I may get back. If I should be going for any length of time, I'll come and say goodbye, of course, wherever you are."

"And Ella; what does she say to your running away from her?" asked Nellie, speaking with a forced cheerfulness.

Arthur's brow darkened.

"Ella and I have differed; I suppose you may say that we've quarrelled. She is like your husband, Nellie," he spoke a little bitterly. "She takes the common-sense view; she thinks that the colonies have been specially designed by Providence for the reception of unwelcome relations, and that Fred should be shipped off to the remotest of them with as much speed and secresy as possible."

"What a shame!" cried Nellie, looking up with burning cheeks.

"She did not know him as we knew him; we must make allowance for her point of view. She never heard of him except as the cousin who must not be mentioned, unless with bated breath by way of warning. I dare say Mr. Coates prayed for him as a reprobate at family worship. He was not a brother to Ella as he was to us."

"I can't bear to think of Ella, there—in aunt Helen's place; it makes me unhappy every time I go to the house."

"She is very good to uncle Joseph and to me, and you must try to like and understand her, Nellie"—he let his hand fall on his sister's shoulder—"for you know she has promised to be my wife."

"Yes," said Nellie, and she sighed. Arthur did not sigh, but there was no ring of a lover's joy and hopefulness in his tone.

